# Effect of age on the diagnostic efficiency of HbA1c for diabetes in a Chinese middle-aged and elderly population: The Shanghai Changfeng Study

**DOI:** 10.1371/journal.pone.0184607

**Published:** 2017-09-08

**Authors:** Li Wu, Huandong Lin, Jian Gao, Xiaoming Li, Mingfeng Xia, Dan Wang, Qiqige Aleteng, Hui Ma, Baishen Pan, Xin Gao

**Affiliations:** 1 Department of Endocrinology and Metabolism, Zhongshan Hospital, Fudan University, Shanghai, China; 2 Fudan Institute for Metabolic Diseases, Shanghai, China; 3 Research Center on Aging and Medicine, Fudan University, Shanghai, China; 4 Department of Clinical Nutrition, Zhongshan Hospital, Fudan University, Shanghai, China; 5 Department of Laboratory Medicine, Zhongshan Hospital, Fudan University, Shanghai, China; Shanghai Diabetes Institute, CHINA

## Abstract

**Background and aims:**

Glycated hemoglobin A1c (HbA1c) ≥6.5% (or 48mmol/mol) has been recommended as a new diagnostic criterion for diabetes; however, limited literature is available regarding the effect of age on the HbA1c for diagnosing diabetes and the causes for this age effect remain unknown. In this study, we investigated whether and why age affects the diagnostic efficiency of HbA1c for diabetes in a community-based Chinese population.

**Methods:**

In total, 4325 participants without previously known diabetes were enrolled in this study. Participants were stratified by age. Receiver operating characteristic curve (ROC) was plotted for each age group and the area under the curve (AUC) represented the diagnostic efficiency of HbA1c for diabetes defined by the plasma glucose criteria. The area under the ROC curve in each one-year age group was defined as AUCage. Multiple regression analyses were performed to identify factors inducing the association between age and AUCage based on the changes in the β and *P* values of age.

**Results:**

The current threshold of HbA1c (≥6.5% or 48mmol/mol) showed low sensitivity (35.6%) and high specificity (98.9%) in diagnosing diabetes. ROC curve analyses showed that the diagnostic efficiency of HbA1c in the ≥75 years age group was significantly lower than that in the 45–54 years age group (AUC: 0.755 *vs*. 0.878; *P*<0.001). Pearson correlation analysis showed that the AUCage of HbA1c was negatively correlated with age (r = -0.557, *P* = 0.001). When adjusting the red blood cell (RBC) count in the multiple regression model, the negative association between age and AUCage disappeared, with the regression coefficient of age reversed to 0.001 and the *P* value increased to 0.856.

**Conclusions:**

The diagnostic efficiency of HbA1c for diabetes decreased with aging, and this age effect was induced by the decreasing RBC count with age. HbA1c is unsuitable for diagnosing diabetes in elderly individuals because of their physiologically decreased RBC count.

## Introduction

Over the past three decades, the prevalence of diabetes worldwide has constantly increased, especially in the elderly population [1, 2]. Previous epidemiologic studies have shown that approximately 60% of people with diabetes are undiagnosed [3, 4], indicating the low awareness of diabetes and urgency to develop more efficient approaches to improve diabetes diagnosis. The special requirements for fasting plasma glucose (FPG) and the oral glucose tolerance test (OGTT) make them inconvenient to use in clinical practice. The HbA1c level reflects glucose homeostasis over the preceding 2–3 months and is convenient to detect without regard to the time; thus, it is superior to the FPG or 2-hour post-load plasma glucose (2-h PG) as an indicator of the average plasma glucose level [5]. With the establishment of international standardized measurements for HbA1c [6], the International Expert Committee recommended a HbA1c level ≥6.5% for the diagnosis of diabetes as it has been shown to be strongly correlated with diabetic retinopathy [7]. The American Diabetes Association (ADA) 2010 and World Health Organization (WHO) 2011 guidelines also recommended a HbA1c level ≥6.5% as the diagnostic criterion for diabetes [8, 9].

However, whether to generalize HbA1c as a diagnostic criterion for diabetes in the entire population remains controversial because of the influence of factors such as age [10–12], race [13–15] and certain diseases [16, 17]. Existing studies have shown that HbA1c demonstrates a limited sensitivity in detecting diabetes in the elderly population [10, 18]. Although age has been reported to be an independent factor affecting the HbA1c levels [19–22], limited literature is available regarding the influence of age on the HbA1c as a criterion for diagnosing diabetes. Some studies have reported that the diagnostic efficiency of HbA1c for diabetes varied in different age groups [23–25]. However, most of these studies were hospital-based and included subjects undergoing health check-ups, and the results could not be generalized to the general population. Whereas the community- or population-based cohort studies were very few. More importantly, existing studies did not explain why age affects the diagnostic efficiency of HbA1c for diabetes.

Based on the above, we conducted a cross-sectional study in a community-based Chinese population to investigate whether age affects the diagnostic efficiency of HbA1c for diabetes, and if so, what induces the age effect.

## Materials and methods

### Subjects

**Fig 1:**
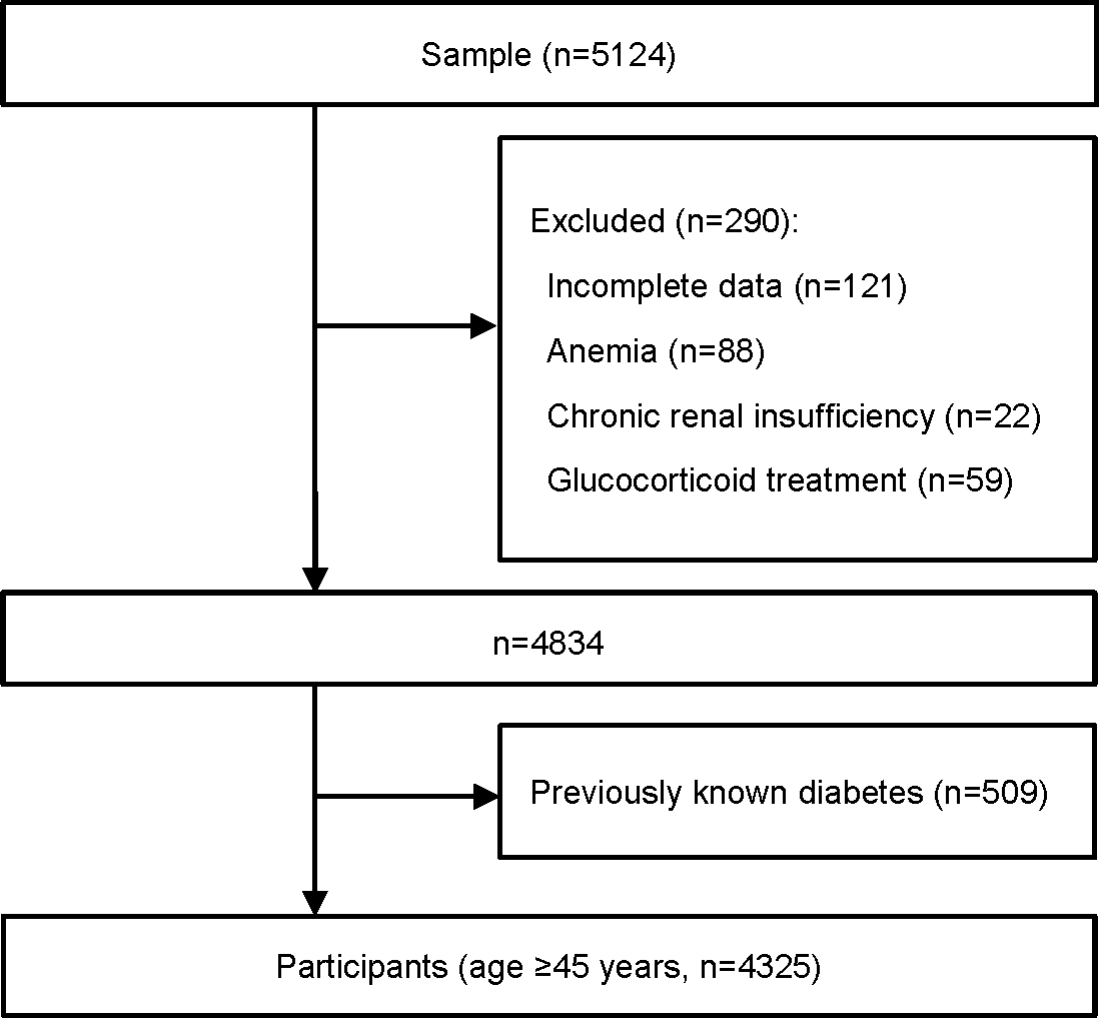
Flow diagram of the recruitment of participants.

From July 2010 to December 2012, 5124 subjects aged ≥45 years were enrolled in the Shanghai Changfeng Study, which is a community-based prospective cohort study researching chronic diseases among the middle-aged and elderly population [26, 27]. Subjects with following diseases or conditions were excluded successively: incomplete data (n = 121), anemia (defined as hemoglobin level <120g/L in male or <110g/L in female, n = 88), chronic renal insufficiency (defined as an estimated glomerular filtration rate <60 mL/min/1.73m^2^ for more than three months [28]mL/min/1.73 m2 for more than 3 months mL/min/1.73 m2 for more than 3 months mL/min/1.73 m2 for more than 3 months mL/min/1.73 m2 for more than 3 months,,,,, n = 22), glucocorticoid treatment (recent three months, n = 59), and previously known diabetes (defined as using hypoglycemic agents or self-reported prior known diabetes, n = 509). Participants with acute illness were asked to visit our study after recovery. Eventually, 4325 participants were included for analyses (Fig 1). All participants signed the written informed consent when entering the study, which was approved by the ethical committee of Zhongshan Hospital affiliated to Fudan University.

### Data collection

Each participant underwent a standard questionnaire by trained investigators to obtain information about the medical history and lifestyle. They were instructed not to alter their diet and physical activity for at least 3 days before the test and were requested to refrain from eating from 9:00 P.M. until the next day’s blood examination. Height and weight were measured with the participants wearing only light clothes and without shoes. BMI was calculated as the weight in kilograms divided by the square of height in meters (kg/m^2^).

After a fasting period of at least 10 hours, the fasting blood samples were collected for the measurement of the fasting plasma glucose (FPG), red blood cell (RBC) count, hemoglobin (Hb), serum creatinine and HbA1c levels. A 75-g OGTT was performed for all participants. The venous blood samples were drawn two hours after the glucose ingestion and were centrifuged immediately, followed by detection within two hours. Plasma glucose was detected using a hexokinase reagent kit (Roche, Swiss) and the Hitachi 7170 analyzer (Hitachi, Japan). The intra- and inter-assay coefficients of variation were <1.07% and <3.52%, respectively. Plasma glucose values for diagnosing diabetes were identified according to the OGTT-based WHO 2011 criteria [9]: FPG ≥7.0mmol/l (126mg/dl), or 2-h PG ≥11.1mmol/l (200mg/dl). HbA1c was measured by high performance liquid chromatography (HPLC) (BIO‐RAD II TURBO), which was certified by the National Glycated Hemoglobin Standardization Program (NGSP). The intra- and inter-assay coefficients of variation for HbA1c were <0.5% and <5%, respectively. The reference range for HbA1c was 4.0–6.0%. The RBC count and Hb were analyzed within 2 hours after venipuncture using an automatic blood counter (Sysmex XE-2100, Japan; coefficient of variation <3.0%). The serum creatinine level was measured using an auto-analyzer (Hitachi 7600; Hitachi, Tokyo, Japan) and standard method. The estimated glomerular filtration rate (eGFR) was calculated using the Modification of Diet in Renal Disease (MDRD) Study formula [28] as follows: 186.3 × [serum creatinine (mg/dL)]^-1.154^ × (age)^-0.203^ × (0.742 if female).

### Statistical analysis

Normally distributed continuous variables were presented as the means ± SD and the others as medians with the interquartile range given in parentheses or number of subjects in each group with percentages in parentheses. One-way ANOVA test was used for comparisons of continuous data among the groups, whereas the Chi-squared test was used for comparisons of categorical variables. Participants were stratified by age. The ROC curve was plotted in each age group and the area under the curve (AUC) represented the diagnostic efficiency of HbA1c for diabetes defined by either FPG or 2-h PG. The area under the ROC curve in each one-year age group was defined as AUCage. Pearson correlation analysis was performed to analyze the association between age and AUCage. Multivariate regression analyses were performed to identify the factors that induce the association between age and AUCage. SPSS version 19.0 was used for all analyses except the ROC curve, which was plotted by MedCalc software. A *P* value <0.05 was considered statistically significant.

## Results

### Characteristics of participants

This study sample had a mean age of 63.1 years with 42.1% males. Participants were stratified into four groups according to age (45–54, 55–64, 65–74, and ≥75 years). As shown in Table 1, with the increase in age, the levels of BMI, FPG, 2-h PG and HbA1c increased; conversely, the levels of eGFR, Hb and RBC count decreased with age (all *P* for trend <0.001).

**Table 1:** Characteristics of participants stratified by age.

	Total (n = 4325)	45–54 years (n = 995)	55–64 years (n = 1691)	65–74 years (n = 1007)	≥75 years (n = 632)	*P* for trend
Age (years)	63.1±9.6	51.9±2.1	59.8±2.9*	69.7±3.0*	79.1±3.2*	<0.001
Male (n, %)	1821(42.1%)	376(37.8%)	691(40.9%)	462(45.9%)*	291(46.0%)*	<0.001
BMI (kg/m^2^)	24.1±3.3	23.7±3.2	24.2±3.2*	24.4±3.4*	24.2±3.4*	<0.001
FPG (mmol/L)	5.36±1.07	5.24±1.03	5.35±1.08*	5.44±1.05*	5.46±1.11*	<0.001
2-h PG (mmol/L)	7.70±3.36	6.90±3.05	7.47±3.10*	8.28±3.24*	8.65±4.19*	<0.001
HbA1c (%)	5.70±0.63	5.60±0.62	5.68±0.64*	5.75±0.57*	5.81±0.65*	<0.001
Hb (g/L)	140.8±12.8	142.2±13.2	141.3±12.5	140.7±12.9*	137.4±12.5*	<0.001
RBC count (10^12^/L)	4.65±0.42	4.74±0.40	4.67±0.41*	4.62±0.42*	4.50±0.43*	<0.001
eGFR (mL/min/1.73m^2^)	94.1±19.0	102.6±118.5	97.0±17.4*	88.8±17.2*	81.6±17.8*	<0.001
Prevalence of diabetes ^a^ (%)	13.8%	8.2%	12.3%	17.9%	20.3%	<0.001
Prevalence of diabetes ^b^ (%)	14.8%	9.1%	12.9%	19.6%	21.5%	<0.001

Data are expressed as means ± standard deviations or the number of subjects in each group with percentages in parentheses.

*P<0.05 *vs*. 45–54 years age group.

^a^ Diabetes diagnosed by the FPG or 2-h PG according to the OGTT-based WHO 2011 criteria

^b^ Diabetes diagnosed by either plasma glucose criteria or HbA1c ≥6.5% (48 mmol/mol).

BMI, body mass index; FPG, fasting plasma glucose; 2-h PG, 2-hour post-load plasma glucose; HbA1c, glycated hemoglobin A1c; Hb, hemoglobin; RBC, red blood cell; eGFR, estimated glomerular filtration rate

Among the 4325 participants without known diabetes, the prevalence of diabetes was 13.8% based on the plasma glucose criteria; when the HbA1c criterion was added, the prevalence of diabetes increased to 14.8%. Moreover, the prevalence of diabetes rose with age, 8.2% (9.1% based on either plasma glucose criteria or HbA1c criterion), 12.3% (12.9%), 17.9% (19.6%), and 20.3% (21.5%) among persons who were 45–54, 55–64, 65–74, and ≥75 years of age, respectively.

### Diagnostic efficiency of HbA1c for diabetes in different age groups

**Fig 2:**
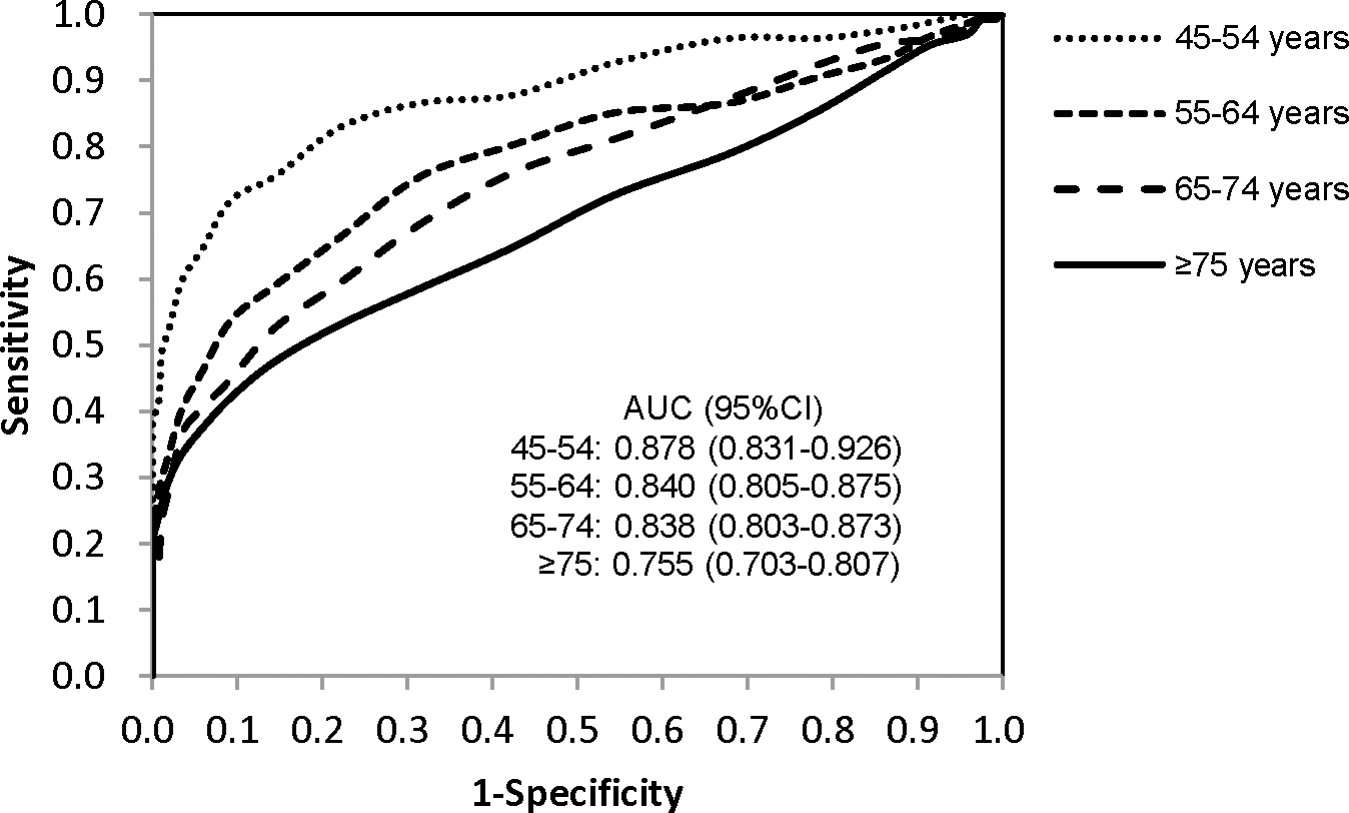
ROC curves of HbA1c for diagnosing diabetes in different age groups. The areas under the curve (AUCs) were 0.878, 0.840, 0.838 and 0.755, respectively with 10 years incrementally increased from 45 years to ≥75 years.

Table 2 shows the sensitivity, specificity, Youden index, positive predictive value (PPV), and negative predictive value (NPV) of the HbA1c threshold 6.5% for diagnosing diabetes in the different age groups. In the total population, the sensitivity and specificity of HbA1c ≥6.5% were 35.6% and 98.9%, respectively. With the increase in age, both the sensitivity and specificity were decreased gradually. Although the differences of sensitivities among the different age groups were not significant (*P* for trend = 0.477), the specificity had a significantly decreasing trend (*P* for trend = 0.013). The Youden index in the 45–54, 55–64, 65–74, and ≥75 years age group were 0.407, 0.330, 0.346 and 0.320, respectively. The ROC curves shown in Fig 2 represent the diagnostic efficiency of HbA1c for diabetes in the different age groups. The AUCs in these age groups were 0.878, 0.840, 0.838 and 0.755, respectively. The diagnostic efficiency of HbA1c in the ≥75 years age group was significantly lower than that in the 45–54 years age group (AUC: 0.755 *vs*. 0.878; *P*<0.001).

**Table 2:** Diagnostic efficiency of HbA1c criterion ≥6.5% in different age groups.

Age-group (years)	Sensitivity (%)	Specificity (%)	Youden index	PPV (%)	NPV (%)
Total	35.6 (31.8–39.6)	98.9 (98.5–99.2)	0.345	83.5 (78.4–87.9)	90.5 (89.6–91.4)
45–54	41.5 (30.7–52.9)	99.2 (98.4–99.7)	0.407	82.9 (67.9–92.8)	95.0 (93.4–96.3)
55–64	33.7 (27.3–40.5)	99.3 (98.8–99.7)	0.330	87.5 (78.2–93.8)	91.4 (90.0–92.8)
65–74	36.7 (29.6–44.2)	97.9 (96.7–98.8)	0.346	79.5 (69.2–87.6)	87.7 (85.4–89.7)
≥75	33.6 (25.5–42.5)	98.4 (96.9–99.3)	0.320	84.3 (71.4–93.0)	85.4 (82.2–88.1)
*P* for trend	0.477	0.013	-	-	-

Values in parentheses are 95% confidence intervals.

Youden index = sensitivity+specificity-1; PPV, positive predictive value; NPV, negative predictive value.

**Fig 3:**
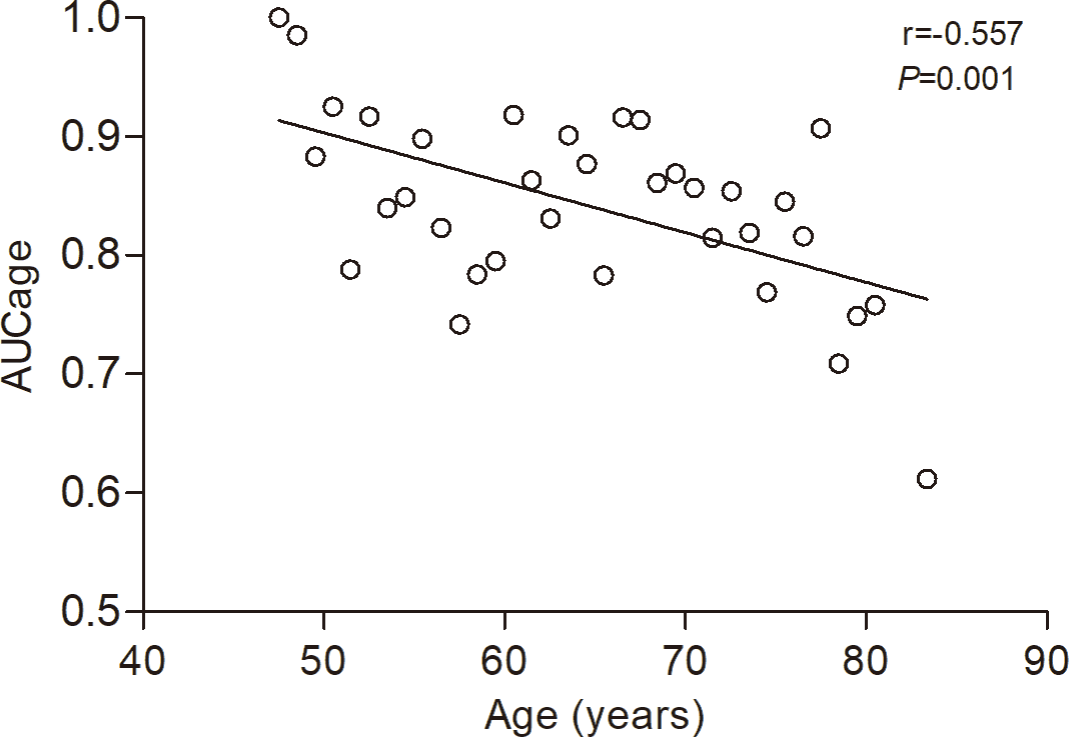
Relationship between the age and AUCage. The AUCage of HbA1c for diagnosing diabetes was negatively correlated with age (r = -0.557, *P* = 0.001). AUCage, area under the ROC curve in each one-year age group (aged 47–81 years, 35 groups in total).

To further evaluate the effect of age on the diagnostic efficiency of HbA1c, we stratified the participants into each one-year age group (aged 47–81 years, 35 groups in total) and the area under the ROC curve for each age group was defined as AUCage. Pearson correlation analysis showed that the AUCage decreased from 47 years to 81 years (r = -0.557, *P* = 0.001) (Fig 3).

### The factor(s) inducing the age effect on the diagnostic efficiency of HbA1c

Except for age, other possible influence factors of AUCage include gender, FPG, 2-h PG, BMI, eGFR, Hb and the RBC count according to the clinical expertise. Univariate analyses showed that age, 2-h PG, eGFR, Hb and the RBC count were all significantly associated with AUCage, while only FPG and the RBC count were independent factors in the multiple stepwise linear regression analysis (Table 3), indicating that the association between age and AUCage was induced by other factors.

**Table 3:** Associations between the possible influence factors and AUCage.

Simple linear regression analyses										Multiple stepwise linear regression analysis	
Variables		Age	Male%	BMI	FPG	2-h PG	RBC count	Hb	eGFR	FPG	RBC count
β value	-0.004		-0.103	-0.023	-0.032	-0.042	0.525	0.02	0.005	0.197	0.647
*P* value	0.001		0.632	0.533	0.774	0.024	<0.001	0.001	0.001	0.042	<0.001

AUCage, the area under the ROC curve in each one-year age group; β, linear regression coefficient; Male%, percentage of males; BMI, body mass index; FPG, fasting plasma glucose, 2-h PG, 2-hour post-load plasma glucose; Hb, hemoglobin; RBC, red blood cell; eGFR, estimate glomerular filtration rate.

To identify the factor(s) that induce the negative association of age and AUCage, we adjusted FPG, 2-h PG, eGFR, Hb and RBC count in the multiple regression model to observe the changes in the β and *P* values of age. First, we separately adjusted each factor and found that the eGFR, Hb and RBC count could all influence the association between age and AUCage. When either eGFR or Hb was added to the model, the negative association between age and AUCage lost significance, with the β value of age decreased to -0.003 and *P* value became >0.05. Moreover, this negative association disappeared when the RBC count was adjusted, with a β value of 0.000 and a *P* value of 0.877 (Table 4). Considering the interactions among these factors, we further successively adjusted them in multiple regression models. As shown in Table 5, when FPG and 2-h PG were simultaneously adjusted, the negative association between age and AUCage was still significant with a β value of -0.007 and *P* value of 0.004. Further adjusting eGFR, the association between age and AUCage became insignificant (*P* = 0.359) and weakened gradually after successively adjusting the Hb and RBC count (β value decreased and *P* value increased gradually). When adjusting the RBC count, the negative association between age and AUCage disappeared, with the β value of age reversed to 0.001 and *P* value increased to 0.856.

**Table 4:** Changes in the β and *P* values of age for AUCage after adjusting FPG, 2-h PG, eGFR, Hb and RBC count separately.

	Age		Adjusted factor	
Models	β value	*P* value	β value	*P* value
Age	-0.004	0.001	-	-
Age + FPG	-0.006	<0.001	0.302	0.008
Age + 2-h PG	-0.007	0.002	0.054	0.110
Age + eGFR	-0.003	0.574	0.001	0.836
Age + Hb	-0.003	0.081	0.003	0.233
Age + RBC count	0.000	0.877	0.495	0.048

Adjusted factors include FPG, 2-h PG, eGFR, Hb and RBC count.

FPG, fasting plasma glucose, 2-h PG, 2-hour post-load plasma glucose; Hb, hemoglobin, RBC, red blood cell; eGFR, estimate glomerular filtration rate; AUCage, the area under the ROC curve in each one-year age group; β, linear regression coefficient.

**Table 5:** Changes in the β and *P* values of age for AUCage after successively adjusting FPG, 2-h PG, eGFR, Hb and RBC count.

Age	FPG	2-h PG	eGFR	Hb	RBC count
-0.004 (0.001)	-	-	-	-	-
-0.006 (<0.001)	0.302 (0.008)	-	-	-	-
-0.007 (0.004)	0.290 (0.035)	0.006 (0.880)	-	-	-
-0.006 (0.359)	0.294 (0.039)	0.004 (0.927)	0.001 (0.885)	-	-
-0.005 (0.399)	0.278 (0.063)	0.004 (0.921)	0.001 (0.917)	0.003 (0.699)	-
0.001 (0.856)	0.283 (0.042)	0.020 (0.604)	0.005 (0.416)	-0.024 (0.083)	0.988 (0.019)

Data are expressed as β values with *P* values in parentheses. Adjusted factors include FPG, 2-h PG, eGFR, Hb and RBC count.

FPG, fasting plasma glucose, 2-h PG, 2-hour post-load plasma glucose; Hb, hemoglobin; RBC, red blood cell; eGFR, estimate glomerular filtration rate; AUCage, the area under the ROC curve in each one-year age group; β, linear regression coefficient.

## Discussion

In the present study, we demonstrated that the diagnostic efficiency of HbA1c for diabetes decreased with aging, and this was induced by the decreasing RBC count with age. To our knowledge, this is the first study from China explaining why the diagnostic efficiency of HbA1c decreased with age.

Although the prevalence of diabetes worldwide has constantly increased in recent years [1, 2], the disease remains underdiagnosed. Existing studies have shown that approximately 60% of diabetes cases are undiagnosed [3, 4]. In this study, 54.0% (598/1107) of diabetes cases were undiagnosed before the survey, which was similar to that in previous studies. The delay in the diagnosis of diabetes could result in a higher risk of developing diabetic complications, which have become the leading cause of impairment in humans [29]. Thus, more efficient approaches for diagnosing diabetes are urgently needed to improve the prognosis of patients with diabetes. HbA1c is superior to the FPG or 2-h PG as an indicator of the average plasma glucose level [5]. Bennett et al [30] reported that HbA1c was similar or superior to the FPG in screening for or diagnosing diabetes. Our results showed that when either the plasma glucose criteria or HbA1c criterion was adopted, the prevalence of diabetes increased from 13.8% to 14.8% (increased by 7.2%). Adding the HbA1c criterion could increase the diagnostic rate of diabetes.

However, HbA1c ≥6.5% was not recommended as a diagnostic criterion for diabetes in China, not only due to the lack of standardized HbA1c measurements but also because of other reasons such as the influence of age on the diagnostic efficiency of HbA1c. Recent studies have shown that the diagnostic efficiency of WHO HbA1c criterion (≥6.5%) varied in different age populations. Kramer et al. [10] reported that the sensitivity and specificity of HbA1c criterion ≥6.5% were 44% and 79%, respectively, in the elderly population (mean age, 69.4±11.1 years). Bao Y et al. [11] showed that using the HbA1c cut-off point of 6.5%, the sensitivity and specificity were 50.5% and 98.1%, respectively, in subjects with a median age of 49.4 years (interquartile range 37.9–57.7). However, Lv X et al. [12] showed that the HbA1c threshold of 6.5% was highly specific (93.9%) and sensitive (95.0%) for diagnosing type 2 diabetes among subjects aged 47.8±11.3 years. In the present study, our sensitivity of 35.6% for HbA1c ≥6.5% was lower than most of other studies. This may due to the different demographic and biochemical characteristics between our study and others. For example, compared with the population in the study of Bao et al, our study participants were older and had a higher BMI, HbA1c and plasma glucose levels. The mean age of our participants was 63.1±9.6 years, and 55.8% of them were aged ≥60 years. These differences may explain why we had a lower sensitivity of HbA1c ≥6.5% and suggested that age may affect the diagnostic efficiency of HbA1c for diabetes.

Although age has been reported to be an independent factor affecting the HbA1c levels [19–22], limited literature is available regarding the influence of age on the HbA1c as a criterion for diagnosing diabetes. In this study, we stratified individuals into four age groups with a 10-year interval and found that both the specificity and sensitivity of HbA1c ≥6.5% for diagnosing diabetes decreased with age, the diagnostic efficiency of HbA1c in the ≥75 years age group was significantly lower than that in the 45–54 years age group (AUC: 0.755 *vs*. 0.878; *P*<0.001). These findings were consistent with the previous studies. Huang H et al. [23] reported that HbA1c performed better in individuals aged <60 years than that in individuals ≥60 years. In Lee’s study [25], the AUCs of HbA1c for diagnosing diabetes were 0.827, 0.800, and 0.771 in the <40, 40–64, and ≥65 years age groups, respectively. Moreover, Yang L et al. [24] found that even using the age-specific HbA1c cut-off points, the diagnostic efficiency of HbA1c also decreased with aging. Furthermore, we stratified the individuals with a 1-year interval and plotted ROC curve for each age group, and found that the AUCage decreased from 47 years to 81 years. These findings confirmed that the diagnostic efficiency of HbA1c decreased with age.

However, why the diagnostic efficiency of HbA1c decreased with age has not been explained before. Our results showed that age was negatively associated with AUCage in univariate analysis (β = -0.004, *P* = 0.001) but showed no significance in multivariate analyses, indicating that the association between age and AUCage was induced by other factors. Interestingly, FPG showed no statistical significance in simple regression analysis but was independently associated with AUCage in multiple stepwise regression. This was mainly due to the confounding among variables in univariate analysis; thus, the effect of FPG was weakened or even masked by other variables with a converse effect. However, in multiple regression analysis, these confounding variables were adjusted and the real effect of FPG appeared. To identify the factor(s) that induce the negative association between age and AUCage, we adjusted FPG, 2-h PG, eGFR, Hb and RBC count in the regression model to observe the changes in the β and *P* values of age. As shown in Tables 4 and 5, the negative association between age and AUCage was still significant either the FPG and 2-h PG were adjusted separately or simultaneously. Since eGFR was adjusted, the association between age and AUCage became insignificant (*P* = 0.359) and weakened gradually after successively adjusting the Hb and RBC count. When adjusting the RBC count, the negative association between age and AUCage disappeared and was even reversed. Although eGFR, Hb and RBC count could all affect the association between age and AUCage, the effect of the RBC count was strongest among them. Because eGFR and Hb were not independently associated with AUCage (*P* = 0.885 and 0.699, respectively) in the multivariate regression model and only slightly affect the association between age and AUCage, we think that they are not the factors directly inducing the association between age and AUCage. The cause might be that individuals with obviously low eGFR or Hb were excluded due to chronic renal insufficiency or anemia. However, the RBC count was not only the independent factor associated with AUCage (*P* = 0.019), it also reversed the association between age and AUCage when added in the regression model. Therefore, we believe that the RBC count was the direct factor that induce the negative association between age and AUCage, and the eGFR might play a role in this process. Except for the RBC count, there may have other factors related to the decreased diagnostic efficiency of HbA1c with age, that need to be identified in further studies.

In this study, we also found that the RBC count decreased with age, a finding that was consistent with the results of Ji C, et al. [31]. This was probably related to the decreased secretion of erythropoietin owing to the decline in renal function with aging, because our results showed that the levels of eGFR decreased with age. Moreover, Ferrucci L et al. reported that androgens which stimulate erythropoietin production, decline with aging [32] and can also lead to a decreased RBC count. Another explanation for the reduced RBC with age may be reduced bone marrow production in the elderly [33, 34]. Some experts also proposed that the decreased red cell turnover owing to the decreased clearance with aging could be a potential explanation [19, 22]. Because we have excluded individuals with anemia or chronic renal insufficiency, the decreasing RBC count with age is probably a physiological process. Nevertheless, how the decreased RBC count leads to the decreased diagnostic efficiency of HbA1c is unclear. We put forward two possible explanations. First, the decreased RBC count caused by the decreased cell turnover could result in the increased RBC lifespan with aging, increasing the levels of HbA1c. Second, previous studies have reported that the cellular damage caused by aging, including altered enzyme activity, decreased membrane lipids and increased cell fragility [35–37], promoting the acceleration of hemoglobin glycation. Under these conditions, the HbA1c level could not truly reflect the average blood glucose concentration in the elderly, resulting in the decreased diagnostic efficiency with aging.

A few limitations need to be considered in this study. First, our participants were middle-aged and elderly; thus, the results might not be applied to the entire population. Second, the previously known diabetes was determined according to self-reporting by the participants. Furthermore, because we did not conduct thyroid function detection and spleen ultrasonography or image in the participants, those with other conditions that affect the concentration of HbA1c, including thyroid dysfunction, splenomegaly and splenectomy, could not be identified and excluded; however, their effect is likely to be small overall. Finally, the diagnostic efficiency of HbA1c was determined based on the results of plasma glucose criteria, and the relationship between the HbA1c level and development of retinopathy associated with type 2 diabetes could not be addressed in this study. Thus, we could not offer age-specific HbA1c cut-off points that are comparable with the WHO 2011 HbA1c criterion for diagnosing diabetes.

## Conclusions

In this community-based study, we found that the diagnostic efficiency of HbA1c for diabetes decreased with aging and this was induced by the decreased RBC count with age. Our study reminds clinicians that the impact of age should be considered when using HbA1c for diagnosing diabetes; HbA1c is unsuitable for diagnosing diabetes in the elderly because of their physiologically decreased RBC count, OGTT is still needed to confirm the diagnosis of diabetes. Our findings need to be further validated by well-designed prospective cohort studies in the future.
